# Super-resolution localization photoacoustic microscopy using intrinsic red blood cells as contrast absorbers

**DOI:** 10.1038/s41377-019-0220-4

**Published:** 2019-11-20

**Authors:** Jongbeom Kim, Jin Young Kim, Seungwan Jeon, Jin Woo BAIK, Seong Hee Cho, Chulhong Kim

**Affiliations:** Departments of Creative IT Engineering, Electrical Engineering, Mechanical Engineering, and Interdisciplinary Bioscience and Bioengineering, Pohang University of Science and Technology (POSTECH), 77 Cheongam-ro, Nam-gu, Pohang, Gyeongbuk 37673 Republic of Korea

**Keywords:** Imaging and sensing, Photoacoustics

## Abstract

Photoacoustic microscopy (PAM) has become a premier microscopy tool that can provide the anatomical, functional, and molecular information of animals and humans in vivo. However, conventional PAM systems suffer from limited temporal and/or spatial resolution. Here, we present a fast PAM system and an agent-free localization method based on a stable and commercial galvanometer scanner with a custom-made scanning mirror (L-PAM-GS). This novel hardware implementation enhances the temporal resolution significantly while maintaining a high signal-to-noise ratio (SNR). These improvements allow us to photoacoustically and noninvasively observe the microvasculatures of small animals and humans in vivo. Furthermore, the functional hemodynamics, namely, the blood flow rate in the microvasculature, is successfully monitored and quantified in vivo. More importantly, thanks to the high SNR and fast B-mode rate (500 Hz), by localizing photoacoustic signals from captured red blood cells without any contrast agent, unresolved microvessels are clearly distinguished, and the spatial resolution is improved by a factor of 2.5 in vivo. L-PAM-GS has great potential in various fields, such as neurology, oncology, and pathology.

## Imaging: superior photoacoustic microscopy

Photoacoustic microscopy with significantly enhanced temporal and spatial resolution has been demonstrated by scientists in South Korea. Jongbeom Kim and coworkers from Pohang University of Science and Technology (POSTECH) incorporated a fast galvanometer scanner and custom-made scanning mirror into their photoacoustic microscope. The result is an imaging system that is able to visualize very fine blood vessels (microvasculature) in the ear, eye or brain of mice that would usually be very hard to detect or undetectable. The scheme does not require an exogenous contrast agent as the light absorption from red blood cells is sufficiently strong and operates with scan rates of up 500 Hz and with 2.5 times improved spatial resolution by an agent-free localization approach. Potential applications that could benefit from the system include vascularization studies in the areas of dermatology and oncology.

## Introduction

Photoacoustic imaging (PAI) is a promising biomedical imaging technique based on the photoacoustic (PA) effect^1^, in which ultrasonic waves are induced by the transient thermal expansion of molecules absorbing optical light. The induced ultrasonic waves, called PA waves, are captured by an ultrasound transducer and are used to reconstruct images. A major implementation of PAI called photoacoustic microscopy (PAM) provides high spatial resolution either by focusing optical beams more tightly than acoustic beams in the optical ballistic or quasi-ballistic regime (referred to as optical-resolution PAM, OR-PAM) or by focusing acoustic beams more tightly than the optical beams in the quasi-diffusive regime (referred to as acoustic-resolution PAM, AR-PAM)^2^. Particularly, OR-PAM has a much higher resolution than AR-PAM because an optical beam can be more tightly focused than acoustic waves. In addition, OR-PAM takes advantage of the unique optical absorption contrast without any contrast agent, being superior to conventional optical microscopic techniques. Thus, due to the unique features of OR-PAM, it enables the anatomical, functional, and molecular imaging of animals and humans in vivo in various research fields, such as biology, oncology, neurology, ophthalmology, dermatology, and pathology^3–15^.

For an outperformed OR-PAM system, a high signal-to-noise ratio (SNR), fast temporal resolution, and high spatial resolution need to be achieved. However, trade-offs exist, which make it difficult to design a PAM system to accomplish all three features. To improve the SNR, which is the most important parameter determining the OR-PAM image quality, opto-acoustic combiners or ring transducers have been applied^6,12,13^. They formed coaxial and confocal alignment of the optical excitation beams and the acoustic emission waves in OR-PAM systems. While this configuration achieves high SNR, it is still a challenge to simultaneously achieve high temporal and spatial resolutions. Therefore, if one increases the temporal resolution, the spatial resolution may be degraded, or vice versa.

Many efforts have been made to improve the temporal resolution^4,6,12,16–23^. The maximum temporal resolution of OR-PAM is theoretically limited by the propagation speed of the generated PA waves in tissues. However, technically, the imaging speed relies on the pulse repetition rate (PRR) of the laser and the scanning mechanism of the system. Several fast laser systems have already been developed to achieve the theoretical temporal resolution, but developed scanning mechanisms maintaining the coaxial and confocal alignment for high SNR have not reached their maximum potential and optimal scanning conditions^24^. The typical OR-PAM systems based on mechanical scanning have a cross-sectional B-scan rate of 1 Hz/mm with a lateral resolution of 2.5 μm, a scanning range of 3 mm, 1200 pixels, and a laser repletion rate of 5 kHz, which is too slow for widespread use in clinical applications^16^. To overcome this slow temporal resolution issue, several scanning mechanisms using a fast voice-coil stage, a microelectromechanical system (MEMS) scanner, a hexagon-mirror scanner and a galvanometer scanner have been reported^4,6,12,17–23^. Although voice-coil PAM increases the B-scan rate up to 40 Hz over a range of 1 mm, the scanning rate is still limited by the mass, driving force, and vibration of the voice-coil stage^17^. Thus, it is difficult to enhance the scanning speed further using mechanical scanning. Water-immersible MEMS scanners increase the B-scan rate up to 400 Hz for the 1-axis and 100 Hz for the 2-axis without using any mechanical scanning^4,6,12,18^. However, these water-immersible MEMS scanners become vulnerable during long-term use, and the acquired PA images are quite distorted because of the unstable scanning patterns. A hexagon-mirror scanner realizes a B-scan rate of 900 Hz over a range of 12 mm^22^. However, the quality of images taken at a B-scan rate of 900 Hz is not relatively good due to the wide step size. Furthermore, not only does an unusable zone result in the laser pulse being wasted, but it is also difficult to adjust the scanning range. The galvanometer scanners have been proven to be suitable for high-speed and stable scanning in conventional optical microscopy systems^19–21,25–30^. However, because the galvanometer scanners are susceptible to the water environment, they have typically been used in OR-PAM for optical scanning only in the air^19–21,23^. In this configuration, it is difficult to achieve coaxial alignment between laser and ultrasound. Thus, these OR-PAM systems based on galvanometer scanners suffer from low SNR and/or a narrow field of view (FOV).

For the spatial resolution of OR-PAM, the lateral resolution is determined by optical diffraction, and the axial resolution is determined by the sound speed and the detection bandwidth of the ultrasound transducer^2^. These theoretical figures have been broken by various techniques using the nonlinear PA effect and localization method^31–35^. Nonlinear PA effects have been applied to attain super-resolution using photobleaching or optical saturation methods^31,32^. However, the penetration depths of these approaches are limited to the sub-surface only. More importantly, the temporal resolution of these systems is very low because they adopt a point-by-point detection mechanism with mechanical scanning. This slow scanning comes naturally from a short working distance of a high-NA objective lens, which makes it difficult to position any opto-acoustic scanning component within the working distance. The main reason to position the scanning module between the objective lens and sample is to maintain the coaxial alignment of the laser and ultrasound for high SNR. Therefore, the conventional super-resolution PAM systems have high SNR and superior spatial resolution but low temporal resolution. Instead of playing with hardware components, localization techniques have been applied to accomplish super-resolution imaging in PAI, ultrasound imaging, and fluorescence microscopy^33–38^. The general principle of the localization imaging method is to superimpose the local maxima positions of all localized sources in the sequence of images. Although the localization method is not heavily restricted by the hardware components, it requires exogenous contrast agents such as microspheres or microbubbles, which are undesirable in clinical settings^36^.

Here, we report a novel agent-free localization PAM system with a galvanometer scanner (L-PAM-GS) to first improve the temporal resolution and then the spatial resolution via the localization process. We placed the galvanometer scanner vertically to keep the scanner body outside the water while the mirror part remained inside, achieving a semi-water-immersible galvanometer scanner. This arrangement enables the scanner to steer both the excitation optical beam and emitted acoustic waves simultaneously. With the enhanced galvanometer scanner, we could improve the temporal resolution to a B-scan rate of 500 Hz with a lateral resolution of 7.5 μm under a scanning range of ~2.4 mm, 500 pixels, and a laser repletion rate of 500 kHz. These superior performances in imaging speed enable the study of microvascular anatomy and the functional hemodynamics of small animals and humans in vivo. More importantly, this fast scanning speed allows us to apply the localization method without any contrast agent. This method locally highlights the PA signals generated from red blood cells momentarily captured during the PA imaging experiments, leading to a high resolution with a low optical NA. In this study, we reconstructed one super-resolved image by post-processing 60 frames of conventional PA volume data taken continuously at a volumetric imaging rate of 2.5 Hz. Therefore, the total integration time was 24 s. We demonstrated improvements from 7.5 μm to 0.4–0.7 μm in the lateral resolution and from 33 to 2.5 μm in the axial resolution in vitro. Furthermore, the unresolved microvessels in the conventional OR-PAM images are clearly visualized in the localization PAM images, and the spatial resolution is improved by a factor of 2.5 in vivo. This L-PAM-GS will be used broadly, with prospective applications ranging from neurology to oncology.

## Results

### Agent-free localization photoacoustic microscopy with a galvanometer scanner (L-PAM-GS) and performance benchmark

**Fig. 1:**
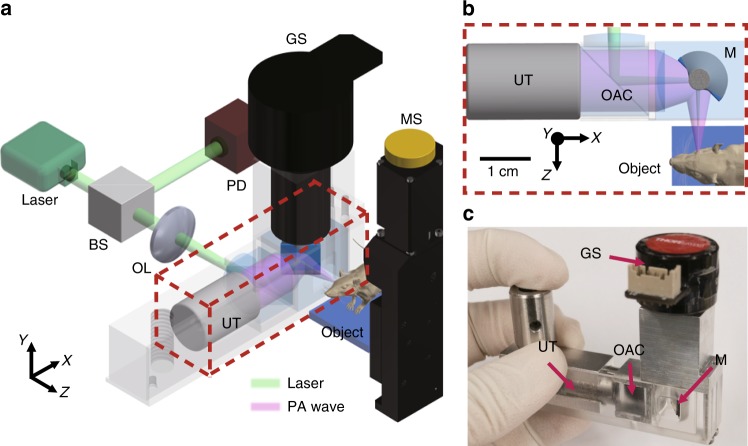
Localization photoacoustic microscopy using a galvanometer scanner (L-PAM-GS). **a** Configuration of the L-PAM-GS system. **b** Close-up view of the scanning part outlined by the red dashed box in **a**. **c** Photograph of the scanning part. BS, beam splitter; OL, objective lens; PD, photodiode; GS, galvanometer scanner; UT, ultrasound transducer; MS, motor stage; OAC, optical-acoustic combiner; M, mirror; FWHM, full width at half maximum.

Figure 1 and Supplementary Fig. S1 show a schematic of the L-PAM-GS system. A nanosecond pulsed laser with a PRR of 800 kHz and an optical wavelength of 532 nm was used to enable ultra-fast and high contrast vascular imaging capabilities. Uniquely, we installed the galvanometer scanner vertically, submerging only the mirror part of the scanner in the water, preventing the scanner from breaking. In addition, we reshaped the mirror part from a flat structure to a half-cylindrical shaft with an aluminum-coated silicon wafer to minimize drag (i.e., fluid resistance), which is proportional to the dynamic pressure projected on a plane perpendicular to the direction of motion in the water environment (Supplementary Fig. S2). Based on computational-fluid-dynamics (CFD) simulation results, the maximum pressure applied to the half-cylindrical mirror part is two times less than that applied to the flat one (Supplementary Fig. S3 and Supplementary Movie 1). The average pressure applied to the half-cylindrical mirror part is four times less because of the larger area of the half-cylindrical mirror part. Our new L-PAM-GS system can achieve a maximum B-scan rate of 650 Hz in water, but stability is guaranteed up to a B-scan rate of 500 Hz. A slow-motion video (Supplementary Movie 2) shows the underwater operation of the galvanometric mirror system at a frequency of 100 Hz. The maximum imaging range (*x*-axis) of the scanner at the 500 Hz B-scan rate was 2.4 mm, and the maximum mechanical scanning range (*y*-axis) was 26 mm. The opto-acoustic combiner was used to align the optical excitation beams and the emitted PA waves coaxially and confocally to increase the SNR. The SNR was calculated to be 35.6 dB in the PA image of a mouse ear in vivo, which is comparable to previously reported results^4,18^. We measured the spatial resolutions of our system by photoacoustically imaging a patterned microstructure and carbon fiber for lateral and axial resolutions, respectively (Supplementary Fig. S4 and Supplementary Text). The optical NA was 0.039, and the lateral resolution was measured to be 7.5 μm, which is very close to the theoretical lateral resolution limit of 7.0 μm^2^. The measured axial resolution was 33.0 μm. This axial resolution matched well with the previously reported resolution of 35.0 μm and the theoretical axial resolution limit of 32.2 μm for a transducer with a center frequency of 50 MHz and a −6 dB bandwidth of 82%^2,39^.

### Microvascular imaging of small animals and humans in vivo

**Fig. 2:**
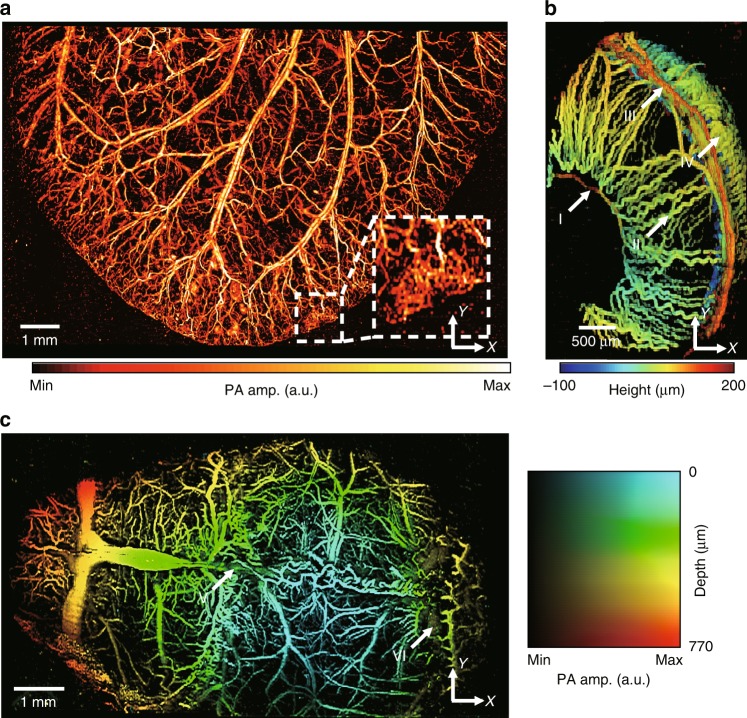
Photoacoustic (PA) images of microvasculatures in small animals in vivo. **a** Wide-FOV PA MAP image of a mouse ear. The region including a capillary bed is outlined by the white dashed box. **b** Depth-encoded PA image of a mouse eye. Circulus arteriosus major (I), iris (II), circulus arteriosus minor (III) and choroid (IV) blood vessels are highlighted by the white arrows. **c** Wide FOV PA MAP image of a mouse brain with color-encoded depths and amplitudes. Superior sagittal sinus (V) and transverse sinus (VI) are highlighted by the white arrows. FOV, field of view; MAP, maximum amplitude projection.

To show the utility of our new system in life science research, we successfully imaged microvasculatures in the ears, eyes, and brains of mice. (Fig. 2). First, we acquired PA MAP images of the mouse ear. The image size of the mouse ear using the L-PAM-GS system was 12.9 mm × 8 mm along the *x*- and *y*-axis, respectively (Fig. 2a). The step sizes were 4.8 and 10 μm along the *x*- and *y*-axis, respectively. Six segmented images, each with a size of 2.4 mm × 8 mm (*x*- and *y*-axis, respectively), were used to synthesize the wider-FOV image. After acquiring one segmented image (2.4 mm × 8 mm along the *x*- and *y*-axis, respectively), we moved the sample stage along the *x*-axis via the manual linear stage, which was attached under the motorized stage (Supplementary Fig. S1b), and then acquired another segmented image. During the image mosaic, 0.3 mm along the x-direction in the adjacent images was overlapped to align each segmented image. Theoretically, under an ideal condition, it takes approximately 9.6 s to obtain all six segmented images (12.9 mm × 8 mm along the *x*- and *y*-axis, respectively), because it takes 1.6 s for one segmented image (2.4 mm × 8 mm along the x- and y-axis, respectively). However, we manually moved the manual stage between each segmented image. Therefore, five manual movements are involved in this case. Therefore, the true total acquisition time for a wide-FOV image was approximately 15 s. The average laser pulse energy used was 200 nJ. The PA MAP image of the mouse ear shows both capillary beds and single capillaries. The capillaries at the edge of the ear generated stronger PA signals compared to the capillaries inside. One possible reason for this difference in the signal signature could be that the skin inside is thicker than that on the edge, which results in more optical scattering.

Next, we imaged the mouse eyes in vivo (Fig. 2b). The FOV of the ocular image is 2.4 mm × 4 mm along the *x*- and *y*-axis, respectively, and the step sizes are 4.8 and 10 μm along the *x*- and *y*-axis, respectively. The acquisition time for one ocular image was 0.8 s. The average laser pulse energy used for ocular imaging was 152 nJ. A random sample consensus machine learning algorithm was applied to accurately segment the ocular structures^11^. The surface-based depth-encoded ocular image visualized the vascular anatomy of eye orbits. Circulus arteriosus major, iris, circulus arteriosus minor and choroid blood vessels were clearly distinguished (highlighted by arrows I, II, III, and IV in Fig. 2b, respectively).

Third, we imaged a wide region (10.1 mm × 6 mm along the *x*- and *y*-axis, respectively) of a mouse brain (Fig. 2c). Before imaging, the mouse’s scalp was removed, but the skull was left intact. Five segmented images with a size of 2.4 mm × 6 mm (*x*- and *y*-axis, respectively) were mosaicked. Similar to the wide FOV ear image of the mouse, each segmented image (acquisition time: 1.2 s) was overlapped by 0.5 mm along the x-direction. The true total acquisition time for a wide-FOV image was ~10 s. The x- and y-axis step sizes were 4.8 and 10 μm, respectively. The average laser pulse energy used for imaging was 200 nJ. The mouse brain image was expressed in a 2D color map showing both depth and amplitude information at once, revealing the detailed angiographic structures of the brain. The superior sagittal sinus and transverse sinus were differentiated from cortical arteries and veins (highlighted by arrows V and VI in Fig. 2c, respectively). In addition, we also quantified the maximum imaging depth in the PA MAP image along the *y*-axis, and the maximum imaging depth for the mouse brain in vivo was ~760 μm (Supplementary Fig. S5a).

**Fig. 3:**
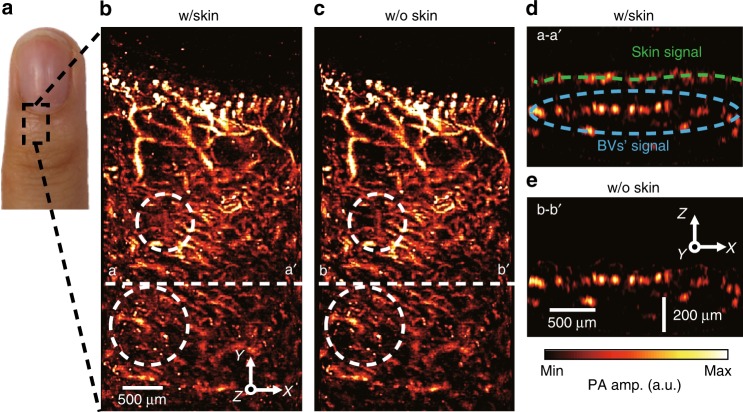
Photoacoustic (PA) images of microvasculatures in humans in vivo. **a** Photograph of a little finger of a volunteer. **b** PA MAP image of the region outlined by the black dashed box in **a**. **c** Skin-removed PA MAP image of **b**. After application to the skin-remover processing, the blood vessels outlined by the white dashed circles in **b**, **c** become clearly visible (also see Supplementary Movie 3). **d**, **e** Cross-sectional PA B**-**mode images of the planes marked by the white dashed lines **d** a–a′ and **e** b–b′. The PA signal from the skin is marked by the green dashed line, and the PA signal from the blood vessels is outlined by the blue dashed circle. The PA signal from the skin was completely removed in the B-mode image of the plane marked by the line b–b′. MAP, maximum amplitude projection; BV, blood vessel.

Finally, we obtained a microvascular PA image of a human fingertip noninvasively (Fig. 3). The region outlined by the black dashed box in Fig. 3a was imaged with our system. Unlike the imaging of small animals, strong PA signals from the skin appeared in the PA MAP images because the skin absorbed much energy from the optical beams. The microvasculature was distinguished relatively weakly in the regions outlined by the white dashed circles in Fig. 3b. Additional post-processing software to remove the skin signals was applied to the 3D volume data before constructing the PA MAP images (Supplementary Fig. S6 and Supplementary Text)^40^. The skin-removed PA MAP image (Fig. 3c) clearly shows the microvessel structures. In the cross-sectional PA B-scan image of the plane marked by the white dashed lines a–a′ in Fig. 3b, the skin signals are clearly revealed (Fig. 3d, green dashed line). However, the skin signals are completely removed in the B-scan image (Fig. 3e) of the plane marked by the white dashed lines b–b′ in Fig. 3c. This difference is even more obvious in the 3D volume rendered movie (Supplementary Movie 3). The FOV of the human cuticle image was 2.4 mm × 4.4 mm, and the step sizes were 4.8 and 5 μm along the *x*- and *y*-axis, respectively. The acquisition time for the human cuticle image was 2 s. The average laser pulse energy used for imaging was 200 nJ. Our system could image the microvasculatures up to ~560 μm in the human cuticle (Supplementary Fig. S5b).

### High-speed PA imaging of hemodynamics in a mouse ear

**Fig. 4:**
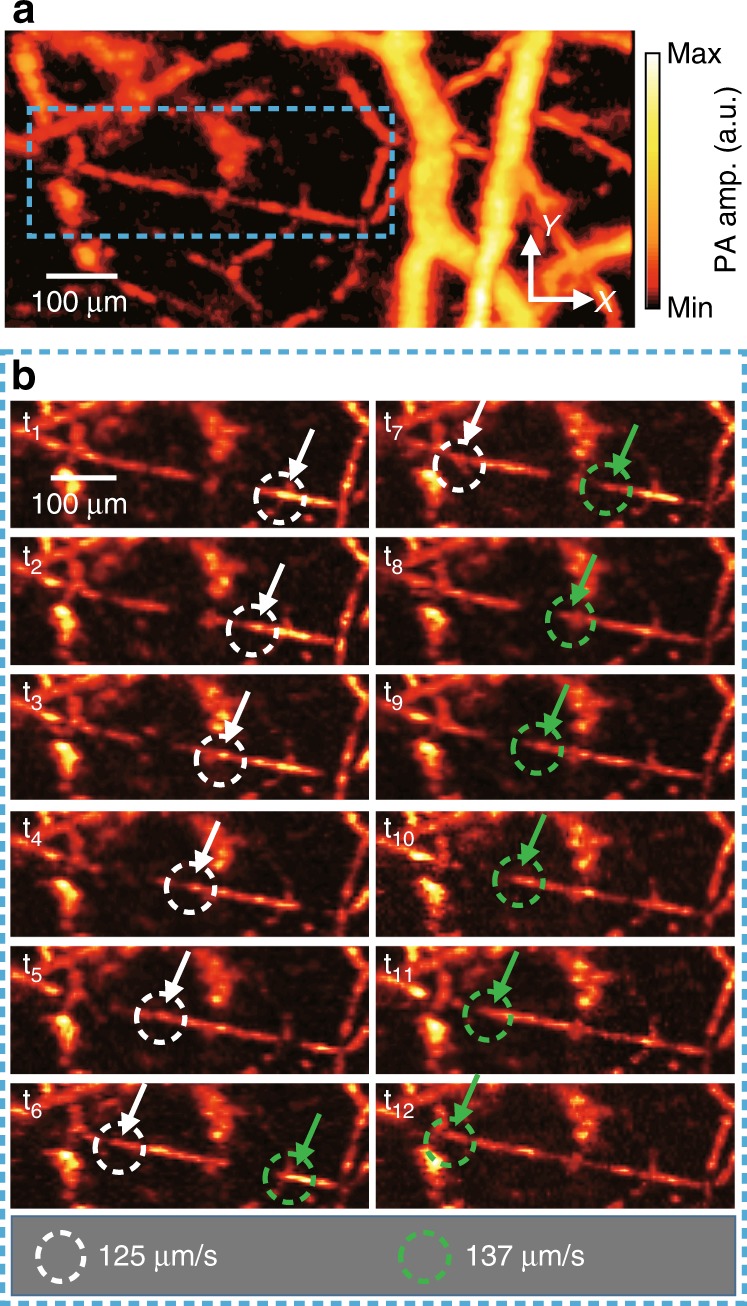
Photoacoustic (PA) monitoring of hemodynamics in a mouse ear. **a** Representative single-shot PA MAP image of the mouse ear. **b** Series of PA images over a time period showing the flow of RBCs (also see Supplementary Movie 4). Close-up views of the region are outlined by the blue dashed boxes in **a**. Flowing red blood cells are highlighted by the white and green dashed circles. The flow rates of the RBCs during the captured period are quantified as shown below. MAP, maximum amplitude projection.

To fully take advantage of the high-speed imaging capability, we monitored the hemodynamics in the microvessels of the mouse ear (Fig. 4 and Supplementary Movie 4). We captured the blood flow and estimated the mean flow rate in the capillaries. A region of 2.4 mm × 0.5 mm (*x*- and *y*-axis, respectively) was imaged repeatedly for 20 s with a volumetric imaging speed of 5 Hz. Both step sizes along the *x*- and *y*-axis were 5 μm. After each PA MAP image was cropped to 890 μm × 420 μm, the image registration process was applied to all PA images to minimize motion artifacts caused by the target movement and the vibration of the scanners (Supplementary Fig. S7). Figure 4a is a representative PA MAP image after the acquisition of a series of images over the acquisition duration. To display the blood flow in the PA MAP image, we traced the PA signals in a capillary in which the amplitude changed. Figure 4b shows a series of PA images in the region outlined by the blue box in Fig. 4a. We estimated the blood flow rate by calculating the change in flow distance of the marked red blood cells (RBCs) (i.e., white and green dotted circles in Fig. 4b) over the image acquisition time. The mean flow rates of the RBCs were estimated to be 125 and 137 μm/s (RBCs highlighted by white and green arrows, respectively). The measured flow rate was similar to the previously reported blood flow rate (e.g., ~100 μm/s) in the vein^41^. Blood flow is also visualized in Supplementary Movie 4.

### Agent-free localization photoacoustic microscopy

**Fig. 5:**
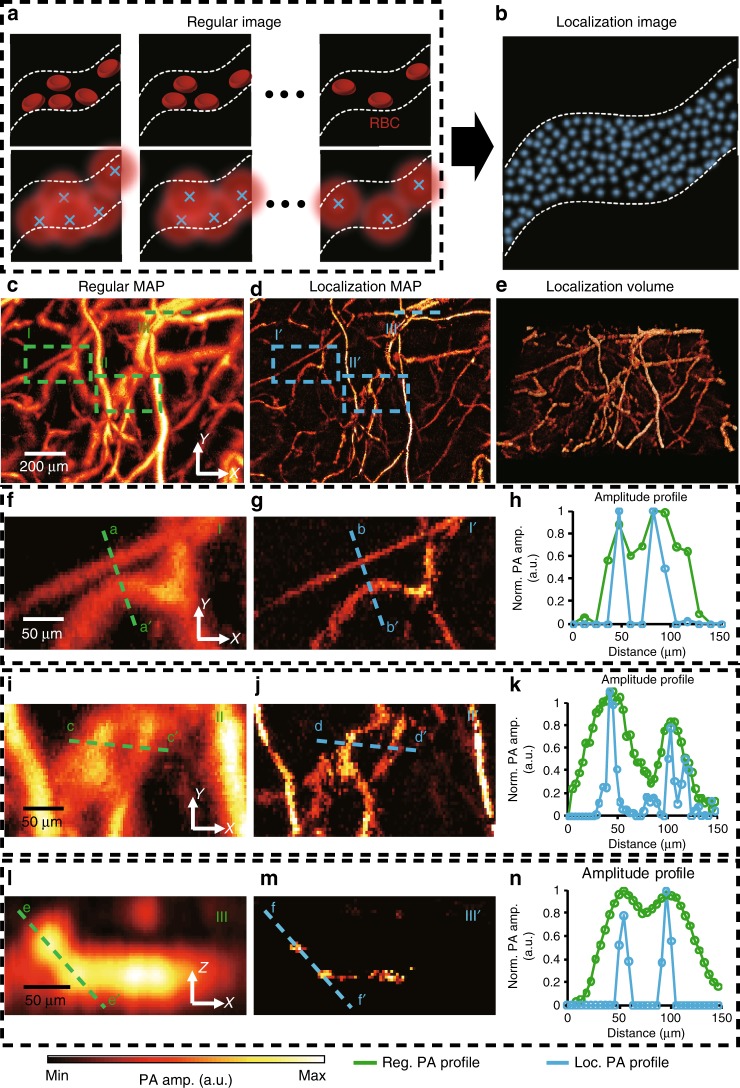
Localization photoacoustic microscopy (PAM) of small animals in vivo. **a**, **b** Principle of agent-free localization PAM. The PA signal from red blood cells is induced by imaging the same microvasculature multiple times. After the PA signals are localized, the localization image is rendered by superimposing all localized positions. **c** Representative PA MAP image of a mouse ear. **d** Equivalent localization image reconstructed with a sequence of 60 frames (also see Supplementary Movie 5). **e** Localization 3D PA image (see also Supplementary Movies 6 and 7). **f**, **g** Close-up views of the regions outlined by the **f** I and **g** I′ dashed boxes in **c** and **d**, respectively. **h** Profiles of the PA amplitude indicated by the green dashed line a-a′ in **f** and of the localization amplitude indicated by the blue dashed line b–b′ in **g**. **i**, **j** Close-up views of the regions outlined by the **i** II and **j** II′ dashed boxes in **c**, **d**, respectively. **k** Profiles of the PA amplitude indicated by the green dashed line c–c′ in **i** and of the localization amplitude indicated by the blue dashed line d–d′ in **j**. **l**, **m** Cross-sectional B-mode images of the region highlighted by the **l** III and **m** III′ dashed lines in **c** and **d**, respectively. **n** Profiles of the PA amplitude indicated by the green dashed line e–e′ in **l** and of the localization amplitude indicated by the blue dashed line f–f′ in **m**. PA, photoacoustic; MAP, maximum amplitude projection.

To confirm the imaging capability of our L-PAM-GS system using the agent-free localization method, we imaged black polystyrene particles in vitro and a mouse ear in vivo (Supplementary Fig. S8 and Fig. 5). In the in vitro experiment (Supplementary Text), the standard deviation of the localized positions of a single particle, determining the enhanced spatial resolution, were 0.4, 0.7, and 2.5 μm (x, y, and z directions, respectively)^33^. We applied our localization method to the label-free PAM of small animals in vivo by localizing the PA signals induced by RBCs. L-PAM-GS is able to capture the RBCs instantaneously based on its fast temporal resolution and high SNR (Fig. 5a). In addition, the PA signals in each frame, generated from the same RBC, can be localized at different positions under the flow condition. The L-PAM-GS image is finally rendered by superimposing all points localized in each frame (Fig. 5b). In this experiment, a sequence of 60 images, each with a size of 1.5 mm × 1 mm (*x*- and *y*-axis, respectively), was obtained, with a volumetric imaging speed of 2.5 Hz (Fig. 5c). Our localization algorithm was applied to each volumetric frame to determine the local maximum points, and the maximum points were superimposed to render localization 2D PA MAP and 3D PA volume images (Fig. 5d, e). Supplementary Movies 5 and 6 illustrate the actual 2D and 3D formations of the improved L-PAM-GS image from the sequence of 60 frames. The rotation movie of the localization volume image also shows the enhanced microvascular structures (Supplementary Movie 7). Supplementary Figure S9 shows the conventional and localization PA MAP images of the mouse ear along the *y*-axis. The imaging depths of both approaches are at least 320 μm, but these are not the maximum imaging depth because the thickness of the mouse ear is too thin to show the maximum imaging depth. To emphasize the enhanced resolution, we enlarged two regions in Fig. 5c (Fig. 5f, i) and corresponding regions in the localization MAP image in Fig. 5d (Fig. 5g, j). The line profiles marked in the magnified images are displayed in Fig. 5h, k. The profiles of the green dashed line a–a′ in Fig. 5f and the blue dashed line b–b′ in Fig. 5g are compared in Fig. 5h. Likewise, the profiles of the green dashed line c–c′ in Fig. 5i and the blue dashed line d–d′ in Fig. 5j are compared in Fig. 5k. As shown in Fig. 5h, the microvessels were clearly resolved into two separate microvessels by L-PAM-GS but were not resolved in the regular PA MAP image. In Fig. 5k, the regular PA profile shows only two microvessels, but the localization profile shows three detached microvessels. We also compared the B-mode images of the region highlighted by the green dashed line in Fig. 5c (Fig. 5l) with its corresponding localization B-mode image (Fig. 5m) to prove the improvement in the axial direction. We compared the profiles of the green dashed line e–e′ in Fig. 5l and the blue dashed line f–f′ in Fig. 5m (Fig. 5n). The blood vessels that could not be resolved in the regular PA B-mode image are well separated in the localization B-mode image, and the profiles confirm this enhancement (Fig. 5n). We compared the B-mode images of the conventional and localization OR-PAM, where a microvessel begins to bifurcate into two, to quantify the improvement in the spatial resolution (Supplementary Fig. S10 and Supplementary Text). These results demonstrate that the improvement in the spatial resolution by a factor of 2.5 was achieved in vivo by an agent-free localization approach. To render a localization PA microvascular image, multiple volumetric data had to be acquired. Thanks to the fast imaging speed (e.g., B-scan rate of 500 Hz) and high SNRs, our system could quickly track the localized PA signals from RBCs by obtaining multiple volumetric data within seconds.

## Discussion

In summary, we developed a fast PAM system that achieves high SNR and high temporal resolution. By installing the modified galvanometer scanner vertically, we successfully steered both the optical beams and the PA waves underwater in the coaxial and confocal formation, leading to a high SNR. The cross-sectional B-scan rate increased to 500 Hz, faster than that of conventional OR-PAM using mechanical scanners, voice-coil scanners, and MEMS scanners^6,12^. Although the hexagon-mirror scanner can achieve a B-scan rate of 900 kHz, it has a wide step size due to the constraints on the fixed scanning range, resulting in poor image qualities^22^. The galvanometer scanner, which generated a linear scanning pattern, provided a more stable scanning pattern than the sinusoidal scanning pattern of the water-immersible MEMS scanners. Taking advantage of the fast speed and stable imaging capability, we acquired wide-FOV images of small animals. We could obtain microvascular images in human cuticles in just 2 s. Moreover, we visualized the flow of RBCs in a mouse ear and quantified the flow rate. Particularly, these outperformed capabilities allow us to apply the localization process to in vivo PAM images without any agent.

In detail, we fabricated an additional half-cylindrical shaft of the scanner to minimize the amount of drag in water. The enhanced galvanometer scanner was placed vertically into the water, immersing only the mirror part. The maximum movement frequency of the scanner in water was 650 Hz. The maximum imaging speed was limited by the performance of the scanner. We developed three main post-processing algorithms: (1) the 3D scan-conversion, (2) the skin-remover, and (3) the image registration algorithms. Our 3D scan-conversion algorithm converts the PA volume data from polar coordinates to Cartesian coordinates. Our algorithm accounts for the scanning geometry (e.g., the range and the phase), similar to general scan-conversion algorithms. Furthermore, we reflected the scanner geometry (e.g., the azimuth, zenith, and elevation tilt) to correct misalignment of the scanner’s mirror. The developed algorithm is not only beneficial in our system; it can potentially be used in PAM systems that use fast angular scanners such as MEMS scanners and galvanometer scanners. Our skin-remover software automatically extracts the skin profile quickly and erases the skin signals in the PA volume data. The skin-removed PA MAP image distinctly showed the microvasculature in humans. This software system can be used effectively in various clinical studies where strong skin signals are present. We also developed a 2D image and 3D volume registration algorithm. The registration algorithm minimizes the motion artifacts in a sequence of the 2D images and 3D volume data. This algorithm can be applied to minimize motion artifacts present in various imaging modalities.

Although L-PAM-GS quantifies the blood flow in a capillary, it is difficult to quantify the flow in arteries and veins. The current imaging speed (e.g., a volumetric imaging speed of 5 Hz) is insufficient to quantify arterial and venous blood flow of several mm/s in a consecutive volume image^42^. To directly quantify the blood flow based on the tracking of RBCs in an artery or vein, the volumetric imaging speed needs to be at least hundreds of Hz. Likewise, the PRR and B-scan rate should be above several tens of MHz and kHz, respectively. To the best of our knowledge, these systems have not yet been developed. Although indirect measurements of blood flow based on correlation are available, new hardware systems for laser systems and scanners must be developed for future works to directly quantify the arterial and venous flow based on tracking^23^. Real-time imaging of the dynamic physiological events in a capillary is important in various preclinical and clinical applications, including hemodynamic response, contrast agent dynamics and transient microcirculatory abnormalities. Because microvascular blood flow is a key parameter of the metabolism in microcirculation, L-PAM-GS has great potential to diagnose and monitor metabolic disorders by visualizing and quantifying microvascular blood flow.

Note that our agent-free localization method affords super-resolved PA images from blood vessels under flow conditions, not from blocked blood vessels. In other words, if the blood vessels are blocked and the RBCs do not flow, such as in strokes, the blood vessels should not be visualized. However, this means that L-PAM-GS can potentially be very useful for noninvasively delineating stroke lesions in vivo. Conventional OR-PAM does not distinguish the lesioned region because the PA signals are still generated from intravascular RBCs even without flow. Our localization technique, on the other hand, does not display the lesioned region (i.e., negative contrast) since there is no flow, and thus, the blocked vessels become distinguishable from normal vessels.

By developing this advanced PAM system, we have provided a promising microscopy tool for future preclinical and clinical studies, including anatomical and functional imaging. Using this system, major blood vessels and angiographic structures in the ears, eyes, and brains of mice were clearly imaged. In particular, the label-free localization method enhanced the spatial resolution of L-PAM-GS. Compared to conventional localization methods, L-PAM-GS does not use any exogenous contrast agent and uses zero-risk RBCs as the absorber to image the microvasculature. We validated the localization algorithm with polystyrene particles in vitro. The agent-free localization imaging approach resolved micro blood vessels, which were not resolved in the regular OR-PAM system. Using the enhanced galvanometer scanner and agent-free signal localization imaging, L-PAM-GS has the potential to monitor peripheral vascular disease, caused in part by impaired microcirculatory function. Furthermore, our system may be a promising tool for early cancer screening by monitoring neovascularization that develops around cancer cells. The L-PAM-GS with high sensitivity to melanin and RBCs can be applied to a new dermoscopy system to diagnose skin conditions and skin diseases, such as skin tumors, warts, and fungal infections. In addition, agent-free L-PAM-GS has potential in neuroscience for monitoring brain hemodynamics and neuron activities as a complementary tool to conventional brain imaging modalities. The L-PAM-GS with deeper penetration depth and lower signal scattering losses than those of fluorescence imaging can quantify the electrophysiological neural activities noninvasively in real time by using various voltage-sensitive dyes. Because of these capabilities of L-PAM-GS, it can also be used noninvasively for the long-term monitoring of optogenetics and detecting neural activities.

## Materials and methods

### Fast agent-free localization photoacoustic microscopy with a galvanometer scanner (L-PAM-GS)

Our L-PAM-GS system mainly consists of an illumination laser, trigger modules, signal acquisition parts, and scanning parts (Fig. 1a–c, and Supplementary Fig. S1). A 532-nm laser source with a PRR of 800 kHz (VPFL-G-10, Spectra-Physics, USA) is used to generate the PA signals. A multifunctional data acquisition board (DAQ, NI PCIe-6321, National Instruments, USA) sends trigger signals to the laser system to fire optical beams. The DAQ sends analog trigonal signals to a galvanometer scanner (GVS001, Thorlabs, USA) and a digital pulse train to a mechanical linear stage (L-406, Physik Instrumente LTD, UK) simultaneously to synchronize the scanning modules. The fired laser pulse is split into two beams by a beam splitter (CM1-BP108, Thorlabs, USA). One split beam strikes a photodiode (PDA36A-EC, Thorlabs, USA), and the other split beam proceeds to illuminate the sample. The current signal generated by the photodiode triggers a digitizer (ATS-9350, Alarzatech, USA), enabling the synchronization of signal acquisition after each laser pulse excitation. The sample beam is focused by an objective lens (LA1131-A, Thorlabs, USA) with a focal length of 50 mm. The objective lens is mounted in a zoom housing (SM1NR1, Thorlabs, USA), which adjusts the optical focal plane along the z-axis. The focused beam passes through an opto-acoustic combiner consisting of a correction lens (NT67-147, Edmund, USA), an aluminum-coated prism (MRA10-G01, Thorlabs, USA), an uncoated prism (PS910, Thorlabs, USA), and an acoustic lens (NT45-382, Edmund, USA) with an acoustic NA of 0.25. In the opto-acoustic combiner, the focused optical beam goes through the correction lens, which corrects optical aberration caused by the acoustic lens. The optical beam is reflected by the aluminum film placed between the two right-angle prisms, and the beam exits the combiner through the acoustic lens. Then, the beam is reflected by the galvanometer scanner mirror in a water tank and illuminates the sample. The mirror module and the acoustic lens of the opto-acoustic combiner are immersed in a water tank, and a window for light and acoustic transmission is sealed with a polyethylene membrane. The generated PA waves are focused by the acoustic lens, allowing the SNR to be further maximized. The focused wave is reflected by the mirror of the galvanometer scanner, travels through the combiner, and is detected by an ultrasound transducer (v214-BC-RM, 50-MHz center frequency, Olympus NDT, USA) with a −6 dB bandwidth of 82%. The signals are amplified by two serially connected amplifiers (ZX60-3018G-S+, Mini-Circuits, 26-dB gain, USA) and then converted into digital signals.

The scanning components are made up of one galvanometer scanner, one motorized linear stage, and two manual translation stages (PT1/M, Thorlabs, USA) to achieve wide-FOV 3D volume imaging (Fig. 1a–c and Supplementary Fig. S1). Each 1D depth-resolved A-scan signal in this system is created by one laser pulse. The fast angular scanning of the galvanometer scanner along the x-axis and the slow linear scanning of the motorized stage along the y-axis accomplish raster scanning. A custom-made sample stage is attached to the motorized stage. Two manual translation stages, fixed below the motorized stage, move the position of the sample stage to take wide-FOV images and adjust the acoustic focus. The position of the sample stage can be moved along the x-axis by the manual stage to obtain the multiple segmented images.

### Simulation and fabrication of a mirror part of a galvanometer scanner

First, we simulated the pressures applied to the mirror parts under oscillation in water according to their geometries (e.g., flat and half-cylindrical) using CFD simulation software (CFD 2019, Autodesk, USA) (Supplementary Fig. S3 and Supplementary Movie 1). The silver mirror part of the flat structure was designed with a width of 10 mm, a height of 8 mm, and a thickness of 1 mm, which mimics the original shape of the mirror in the galvanometer scanner. The half-cylindrical mirror part was designed with a diameter of 8.5 mm and a height of 8 mm and was made from acrylonitrile butadiene styrene copolymer material. Both mirror parts were set to oscillate at an angle of ±8.63° at a speed of 500 Hz. We specifically fabricated a new mirror part of the galvanometer scanner, which consists of an additional shaft and mirror (Supplementary Fig. S2). The new shaft was designed in the form of a half cylinder to minimize the drag in water. The mirror was made with a silicon wafer with a diameter of 10.2 cm. The wafer was coated with 100-nm-thick aluminum layer using an evaporator and then sliced with a grid pattern of 8.5 mm × 6 mm using a dicing saw. One piece of the sliced wafer was attached to the flat surface of the fabricated shaft with an instant adhesive. We replaced the built-in mirror part with the custom-made mirror.

### Photoacoustic imaging of animals in vivo

All animal procedures in all experiments were permitted by the Institutional Animal Care and Use Committee of Pohang University of Science and Technology (POSTECH). All animal experiments were conducted according to the National Institutes of Health Guide for the Care and Use of Experimental Animals. Normal female Balb/c mice (16–30 g, 3–8 weeks old) were prepared for in vivo PA imaging. The mice were initially anesthetized with a 4% isoflurane gas vaporized by inhalation gas (1.0 L/min flow rate), and during imaging, anesthesia was maintained with 1% isoflurane. The mice were placed on a silicon-heating pad on the sample stage to maintain their body temperature during the experiment. To image an ear and eye of a mouse, the hair was removed with a depilatory. We applied ultrasound gel to match the acoustic impedance between animals and the polyethylene membrane. The laser pulse energy of 10 mJ/cm^2^ used for the ear is within the American National Standards Institute (ANSI) safety limit of 20 mJ/cm^2^ for visible light. We calculated the maximum permissible exposure (MPE) for ocular exposure with the ANSI safety standards (Supplementary Materials and Methods)^43^. The maximum permissible single laser pulse energy for a human pupil is 1.0 μJ and 365 nJ for single and multiple imaging, respectively, which are higher than the experimentally used pulse energy of 152 nJ. In imaging a mouse brain, the mouse’s scalp was removed, but the skull was left intact. After the scalp was removed, the vessels on the skull were carefully cleaned with saline solution to acquire the PA signals from only the brain vessels. Ultrasound gel was applied to the skull to prevent the brain tissue and skull from drying out and to match the acoustic impedance between the skull and the water tank. After the mouse was laid sideways on the sample stage, the head was fixed by the support to reduce the motion artifact. The laser energy used was 8.3 mJ/cm^2^.

### Photoacoustic imaging of a human finger in vivo

Imaging of humans was conducted according to protocols approved by the Institutional Review Board of POSTECH. We recruited a subject and provided the subject with complete information regarding the study. The recruited subject signed consent documents to participate in the experiments. Although the used pulsed laser illumination energy of 2.4 mJ/cm^2^ was less than the ANSI safety limit of 20 mJ/cm^2^, the participant wore protective equipment, protective glasses and heatproof clothes to protect their eyes and body against laser light beams. Before imaging a digitus annularis (ring finger), mineral oil was applied to moisten the fingertip, and then an ultrasonic gel was applied. The hand was fixed to the sample stage, and the finger coupled with the ultrasound gel was stuck onto the side of the sample stage. Other motions were prohibited during imaging to reduce motion artifacts. The scan-conversion algorithm was applied to all PA volume data, followed by a skin-removing algorithm to the converted data, to image the vasculature^40^.

### Localization photoacoustic microscopy in vivo

Our localization process is divided into four main steps: (1) the fast acquisition of a sequence of PA volume data, (2) the precise alignment of the PA volume data, (3) the determinant of local maximum points in the volume data, and (4) the rendering of localization 2D or 3D models. In the in vivo localization imaging experiments, the same area of the mouse ear was imaged consecutively. First, a sequence of 3D volume data was aligned via the intensity-based image registration algorithm. The volume data were interpolated to a size of 4x by bicubic interpolation, and the interpolated data were then normalized. The 3D averaging filter was applied to the normalized data in the 3D volume to emphasize local maximum points, and the local maximum positions were then determined in MATLAB. By superimposing multiple positions in the 3D data, localization MAP and localization 3D volume images were reconstructed.

## Supplementary information

Supplementary Information for Super-resolution Localization Photoacoustic Microscopy using Intrinsic Red Blood Cells as Contrast Absorbers

Simulation video of pressures applied to the mirrors with flat and half cylindrical structures.

Slow motion video of scanner movement in water.

3D PA images of human cuticles with and without skin signals.

Hemodynamics in the mouse ear.

Actual formation of the 2D localization PA MAP image.

Actual formation of the 3D localization PA volume image.

Rotation video of the 3D localization PA volume image.

## Data Availability

All data are available within the Article and Supplementary Files or available from the authors upon request.
