# Supplementary material for: Super-resolution localization photoacoustic microscopy using intrinsic red blood cells as contrast absorbers
Source: Light Sci Appl. 2019 Nov 20;8:103. doi: 10.1038/s41377-019-0220-4 (PMC6868204; doi:10.1038/s41377-019-0220-4)
Supplement: Supplementary file 1 — Supplementary Information for Super-resolution Localization Photoacoustic Microscopy using Intrinsic Red Blood Cells as Contrast Absorbers [file 41377_2019_220_MOESM1_ESM.docx]

Supplementary Information for

**Super-resolution Localization Photoacoustic Microscopy using Intrinsic Red Blood Cells as Contrast Absorbers**

Jongbeom Kim, Jin Young Kim, Seungwan Jeon, Jin Woo BAIK, Seong Hee Cho, Chulhong Kim^*^

^*^Corresponding author. Email: chulhong@postech.edu

**This file includes:**

**Supplementary Text**

**Supplementary Materials and Methods**

**Supplementary Fig. S1** Schematic and photograph of the L-PAM-GS system.

**Supplementary Fig. S2** Design of the custom-made mirror part of the galvanometer scanner.

**Supplementary Fig. S3** Simulation result comparing the pressures applied to each geometry.

**Supplementary Fig. S4** Spatial resolution of the L-PAM-GS system.

**Supplementary Fig. S5** *In vivo* conventional PA MAP images of **a** a mouse brain and **b** human cuticle along the y-axis to show the maximum penetration depths.

**Supplementary Fig. S6** Skin removal process.

**Supplementary Fig. S7** Image registration.

**Supplementary Fig. S8** Localization photoacoustic microscopy (PAM) of polystyrene particles *in vitro*.

**Supplementary Fig. S9** *In vivo* **a** conventional and **b** localization PA MAP images of a mouse ear along the y-axis.

**Supplementary Fig. S10** Quantification of the spatial resolution improvement with agent-free localization imaging *in vivo*.

**Movie files:**

**Supplementary Movie 1** Simulation video of pressures applied to the mirrors with flat and half cylindrical structures.

**Supplementary Movie 2** Slow motion video of scanner movement in water.

**Supplementary Movie 3** 3D PA images of human cuticles with and without skin signals.

**Supplementary Movie 4** Hemodynamics in the mouse ear.

**Supplementary Movie 5** Actual formation of the 2D localization PA MAP image.

**Supplementary Movie 6** Actual formation of the 3D localization PA volume image.

**Supplementary Movie 7** Rotation video of the 3D localization PA volume image.

**Supplementary Text:**

**Spatial resolution measurement.**

We measured the spatial resolutions of our system with a patterned microstructure and carbon fiber (Supplementary Fig. S4). The patterned microstructure was imaged to measure the lateral resolution of the L-PAM-GS system with a step size of 0.5 μm along the x-axis. Each maximum amplitude projection (MAP) data across a-a′ marked in Supplementary Fig. S4a was averaged with the neighborhood MAP data values of sixty points along the y-axis. An edge spread function (ESF) was fitted using the averaged MAP data. The full width at half maximum (FWHM) of a line spread function (LSF), the first derivative of the ESF, is defined as the lateral resolution. The optical NA was 0.039, and the lateral resolution was measured to be 7.5 μm (Supplementary Fig. S4c). We also measured the axial resolution by imaging the carbon fiber with a diameter of about 6 μm (Supplementary Figs. S4b, d). The LSF was fitted using cross-sectional data across b-b′ marked in Supplementary Fig. S4b. The FWHM of the LSF is defined as the axial resolution, and the measured axial resolution was 33.0 μm (Supplementary Fig. S4d).

**Skin removal process.**

First, skin location was estimated roughly by a pre-set value in each A-line data (Supplementary Figs. S6b, c). After the skin location was detected in all the A-line data, the 2D skin profile was smoothed by a 2D Gaussian low-pass spatial filter (Supplementary Figs. S6d, e). The two regions, comprising skin signals and blood vessels signals, were separated by adjusting the elevation of the skin profile along the z-axis (Supplementary Figs. S6f, g). Finally, the skin signal region was removed (Supplementary Figs. S6h, i).

**Localization photoacoustic microscopy (PAM) of polystyrene particles *in vitro*.**

We first validated our localization algorithm with black polystyrene particles (Supplementary Fig. S8). Because the particle size (mean diameter, 0.8 μm) was smaller than the regular spatial resolution of L-PAM-GS, the photoacoustic (PA) MAP and cross-sectional images of single particle corresponded to the point spread functions (PSF) of our system (Supplementary Figs. S8a, c). We reconstructed the images as the histogram of the local maximum points of the PSFs for 100 frames of the image sequence (Supplementary Figs. S8b, d). We compared the PSFs with the fitted Gaussian curves, which show the histograms of the x, y and z coordinates of the localized positions (Supplementary Figs. S8e-g). In localization fluorescence microscopy, the localization precision is generally characterized by the standard deviation (SD) of multiple localized positions of a single particle^1^. The localization spatial resolution can be determined by the variability in the localized position of an absorber^2^. The SDs of the multiple localized positions of the same source were 0.4 μm and 0.7 μm (x and y coordinates, respectively). For the axial direction (z coordinate), the SD was 2.5 μm. The discrepancy for the slightly higher values in the y-direction compared to the x-direction is due to the vibration of the motorized stage. The higher SD in the axial direction is caused by jitter in our data acquisition system. Thus, the comparison in Supplementary Figs. S8e-g demonstrates the improvement in spatial resolution achieved with L-PAM-GS compared to regular optical-resolution PAM.

**Quantification of the spatial resolution improvement with agent-free localization imaging *in vivo*.**

We compared the profiles of conventional PA and localization amplitudes in the B-mode images, where microvessels begin to bifurcate into two, to quantify the spatial resolution with agent-free localization imaging (Supplementary Fig. S10). Supplementary Figures S10a and b show representative conventional and its corresponding localization PA MAP images of a mouse ear *in vivo*, respectively. First, we compared the B-mode images of the C1 and C1′ regions highlighted in Supplementary Figs. S10a, b to measure the spatial resolution of localization imaging *in vivo* (Supplementary Figs. S10c, d). In the C1′ region, a microvessel begins to be split into two, and the distance between the two peaks is approximately 20 μm (Supplementary Fig. S10e). Likewise, to measure the spatial resolution *in vivo* of the regular PAM system, we compared the B-mode image of the C2 region in Supplementary Fig. S10a, where a microvessel begins to appear in two, with the corresponding localization B-mode image from the C2′ region in Supplementary Fig. S10b (Supplementary Figs. S10f, g). The distance between the peaks was approximately 50 μm (Supplementary Fig. S10h). These results demonstrate that the improvement in spatial resolution by a factor of 2.5 is achieved by agent-free localization. The measured spatial resolutions in the *in-vivo* experiments are larger than the values measured in the phantom experiments because of the increased focal spot size due to optical scattering, the inability to accurately select the point, where blood vessels begin to separate, and the wide scanning step size.

**Supplementary Materials and Methods:**

**3D scan-conversion algorithm.**

We developed a 3D scan-conversion algorithm to convert PA volume data from the polar coordinate to the Cartesian coordinate. Each B-scan data along the x-axis in the PA volume data is in the polar coordinate since the data is acquired by the angular scanning of the galvanometer scanner. To present the PA volume data in the intuitive form, such as 2D image and 3D volume in the Cartesian coordinate, the scan-conversion algorithm is applied to all volume data. Our algorithm takes into consideration both scanner’s geometry (e.g., the azimuth, zenith, and elevation tilts) and the scanning geometry (e.g., the range and the phase).

**Registration of 2D PA image and 3D volume data.**

To minimize motion artifacts caused by the sample movement and the vibration of the scanner and the motorized stage during the imaging experiments, we used a 2D image and 3D volume registration algorithm. This algorithm uses intensity-based registration and compares intensity patterns in datasets via correlation metrics. The algorithm uses two datasets as inputs. One dataset is referred to as moving dataset, and the other dataset is referred to as target dataset or reference dataset. Both the moving dataset and the target dataset must be of the same dimension in 2D or 3D. The moving dataset is spatially transformed to align with the target dataset. The geometric transformations applied to the moving dataset were a translation, rotation, scale, and shear. In the processing, we first selected a reference dataset among the sequence of a dataset acquired. The selected reference dataset was then used to align all the other dataset.

**Maximum permissible exposure for ocular exposure.**

We calculated the maximum permissible exposure (MPE) for ocular exposure with the American National Standards Institute (ANSI) safety standards^3^. First, the MPE for a single laser pulse is *MPE_sp_* = 5*C_E_* × 10^-7^ = 2.6 × 10^-5^ J cm^-2^, where *C_E_* is a correction factor and is calculated to be 52.0. Secondly the repetitive pulse limit is *MPE_RP_* = *n_single_*^-0.25^ × *MPE_sp_* = (2 × 10^5^)^-0.25^ × 2.6 × 10^-5^ = 2.0 × 10^-6^ J cm^-2^, where *n_single_* = 3 × 10^4^ is the total number of effective pulses during the single imaging. We also considered one cross-sectional scan (B-scan) containing 500 pulses with a PRF of 500 kHz. The MPE for the B-scan pulse train is *MPE_Bscan_* = 1.8*C_E_* × 0.001^0.75^ × 10^-3^ = 5.3 × 10^-4^ J cm^-2^, where 0.001 means the B-scan exposure time of 0.001 s. Thus, the MPE/pulse for the B-scan pulse train is *MPE_Bscan_*/*n_Bscan_* = 1.1 × 10^-6^ J cm^-2^, where *n_Bscan_* = 500 is the number of pulses during B-mode imaging. Next, we consider MPE for the single imaging. Both photochemical and thermal effects are considered. The MPE for the photochemical effects is *MPE_SI_*(*photochemical*) = 100 × *C_B_* ÷ (2 × π ×0.003) = 2.3 × 10^6^ J cm^-2^, where *C_B_* = 10^0.02(532-450)^ = 43.7 is the wavelength correction factor. The MPE for the thermal effects is *MPE_SI_*(*thermal*) = 1.8 × *C_E_* × 0.8^0.75^ × 10^-3^ = 7.9 × 10^-2^ mJ cm^-2^, where 0.8 means the single imaging exposure time of 0.8 s. Thus, the MPE/pulse for the single imaging pulse train is *MPE_SI_*/*n_single_* = 2.0 × 10^-6^ J cm^-2^. For the single imaging, the maximum permissible single laser pulse energy for a human pupil with the diameter of 7 mm is calculated to be *MPE_SI_*(*thermal*) × π × (*D* / 2)^2^ = 1.0 μJ, where *D* is the diameter of a human, which is higher than the experimentally used pulse energy of 152 nJ. Furthermore, we calculated MPE for the multiple imaging, considering that our localization method will be applied to ocular imaging as a future work. The total imaging frame number is assumed to be 60 used for the localization processing. The MPE for the photochemical effects is *MPE_MI_*(*photochemical*) = 100 × *C_B_* ÷ (2 × π × 0.003) = 2.3 × 10^6^ J cm^-2^. The MPE for the thermal effects is *MPE_MI_*(*thermal*) = 1.8 × *C_E_* × 48^0.75^ × 10^-3^ = 1.7 J cm^-2^. Thus, the MPE/pulse for the multiple imaging pulse train is MPE_MI_/*n*_multiple_­­ = 9.5 × 10^-7^ J cm^-2^, where *n_multiple_* = *n­­_single_* × 60 is the total number of effective pulses during the multiple imaging. For the multiple imaging, the maximum permissible single laser pulse energy for the human pupil is calculated to be *MPE_MI_*(*thermal*) × π × (*D* / 2)^2^ = 365 nJ, which is also higher than the used pulse energy of 152 nJ.

**Localization photoacoustic microscopy *in vitro*.**

To explore localization in photoacoustic microscopy, we conducted localization experiments with 0.8-μm-polystyrene-black-dyed microspheres (DSBK005, Bangs Laboratories, Inc., USA). The water-diluted microsphere solution was placed on a glass slide and then was fixed with a polyethylene membrane. The same region was photoacoustically imaged multiple times. A single microsphere was selected manually in a representative PA MAP image, and then the 3D volume data of the selected microsphere was taken out in the full 3D data. The intensity-based image registration algorithm aligns 3D volume data of the microsphere to reduce the displacement caused by the vibration of the mechanical scanning. The local maximum positions were determined in the multiple 3D volume data in MATLAB. We built a 2D histogram by counting the number of the determined positions in each pixel. The profiles of the PA signals and the 2D histogram values were obtained at the same coordinates of the regular PA image and the localization 2D histogram, respectively. Before obtaining the profile of the PA signals, a moving 3 × 3 median filter was applied to the representative PA MAP image of the single microsphere. Piecewise Cubic Hermite Interpolating Polynomial interpolation was applied to the profiles, and the profiles were then fitted by a Gaussian function.

**Supplementary Figures:**


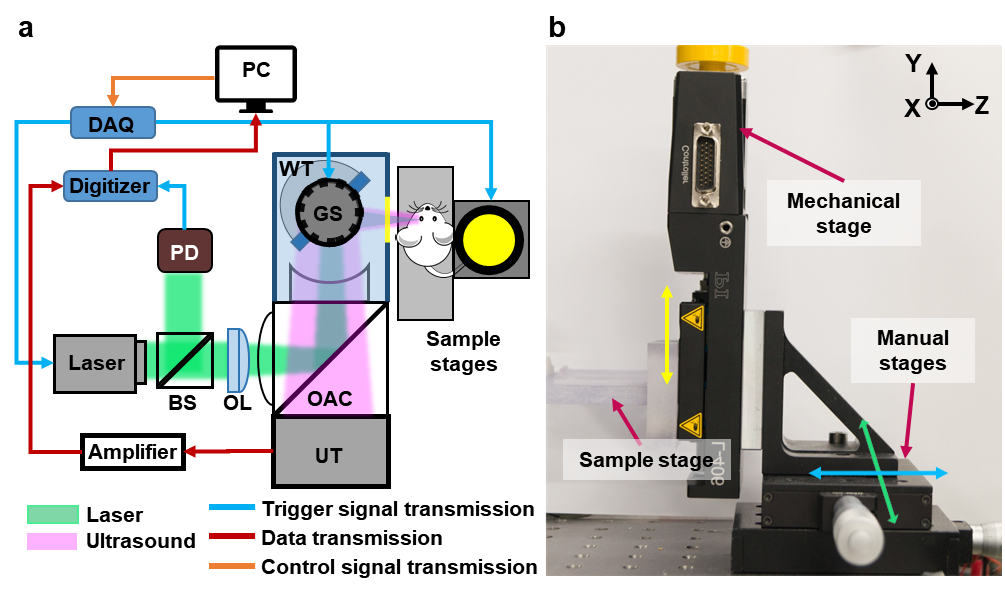


**Supplementary Fig. S1** Schematic and photograph of the L-PAM-GS system. **a** Signal flow diagram of L-PAM-GS. **b** Photograph of sample stages. Yellow bidirectional arrow indicates the direction of movement of the motorized linear stage along the y axis. The manual stages enable the sample stage to move along the directions of the blue and green bidirectional arrows to focus acoustic signals and acquire wide FOV images, respectively. PD, photodiode; BS, beam splitter, OL, objective lens; WT, water tank; GS, galvanometer scanner; OAC, optical-acoustic combiner; UT, ultrasound transducer.

**
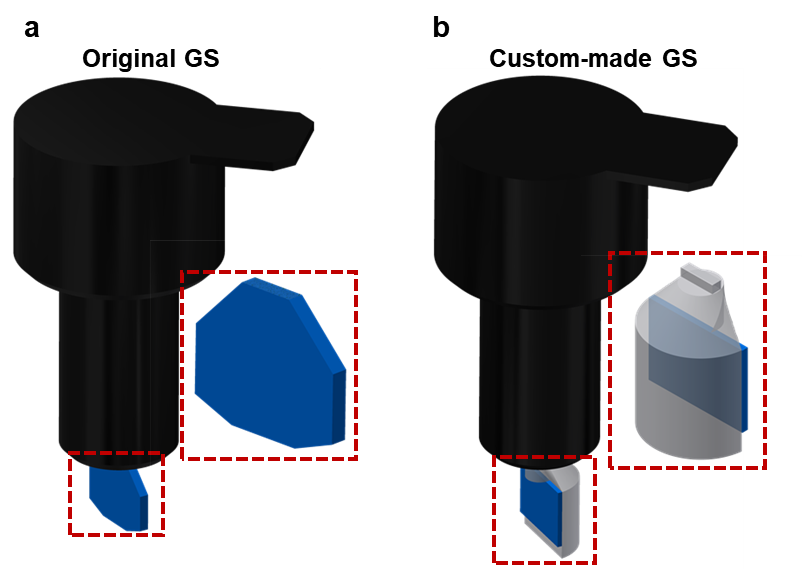
**

**Supplementary Fig. S2** Design of the custom-made mirror part of the galvanometer scanner. **a** GS and the original built-in mirror part. The built-in mirror part of the GS is flat on both sides. **b** GS and the custom-made mirror part. The mirror part consists of an additional half cylinder shaft and a mirror. GS, galvanometer scanner.


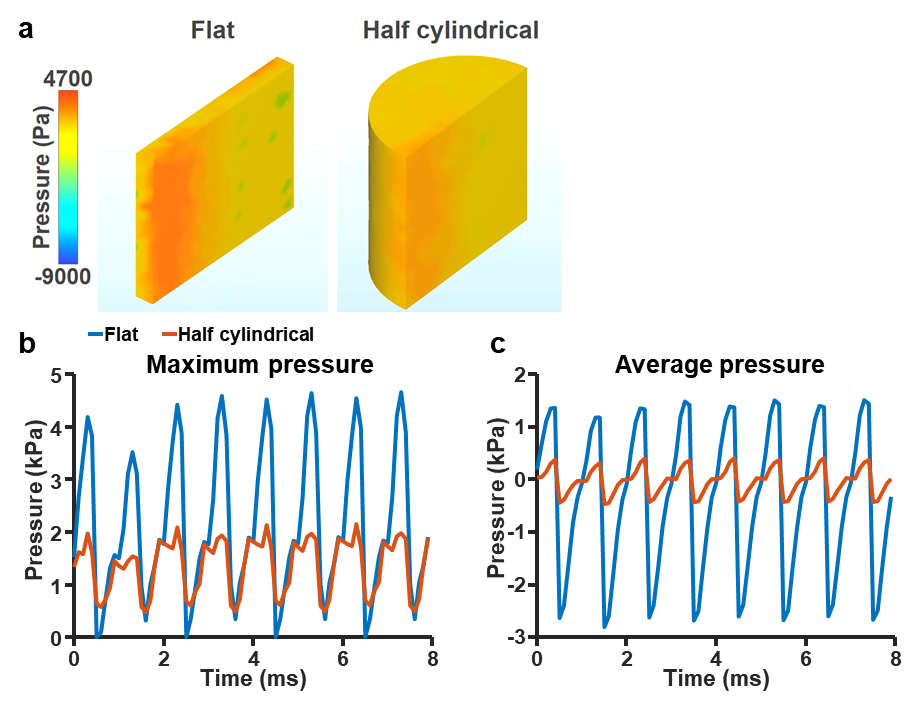


**Supplementary Fig. S3** Simulation result comparing the pressures applied to each geometry. **a** Snapshot of the simulation. **b**, **c** Time courses of **b** maximum and **c** averaged changes on pressure applied to the mirrors with a flat, half cylindrical, and cylindrical structures (also see **Movie S1**).

*
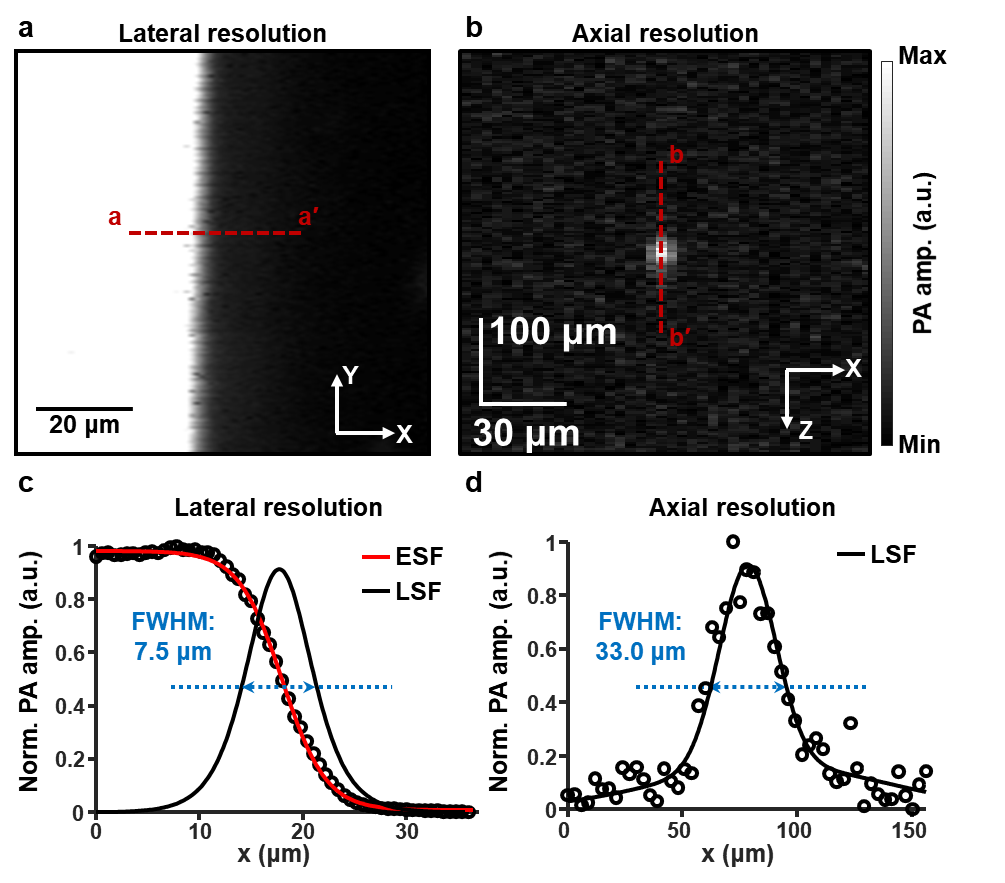
*

**Supplementary Fig. S4** Spatial resolution of the L-PAM-GS system. **a** PA MAP image of the edge of a patterned microstructure. **b** Cross-sectional PA B-scan image of a carbon fiber. **c** Fitted ESF of PA data marked by the line a-a′ in **a** and LSF derived from the first derivative of the ESF. The lateral resolution was measured by the FWHM of the LSF. **d** Fitted LSF of PA data marked by the line b-b′ marked in **b**. The axial resolution was measured by the FWHM of the LSF. PA, photoacoustic; ESF, edge spread function; LSF, line spread function; MAP, maximum amplitude projection.


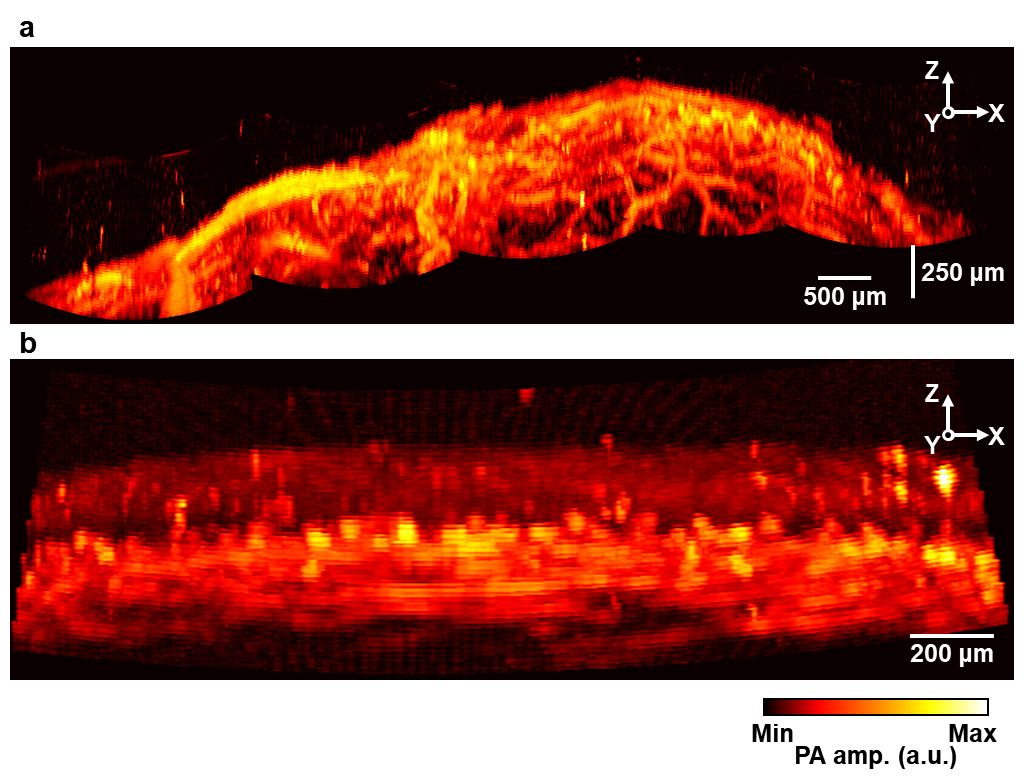


**Supplementary Fig. S5** *In vivo* conventional PA MAP images of **a** a mouse brain and **b** human cuticle along the y-axis to show the maximum penetration depths. The color bar is based on a log scale. PA, photoacoustic; MAP, maximum amplitude projection.


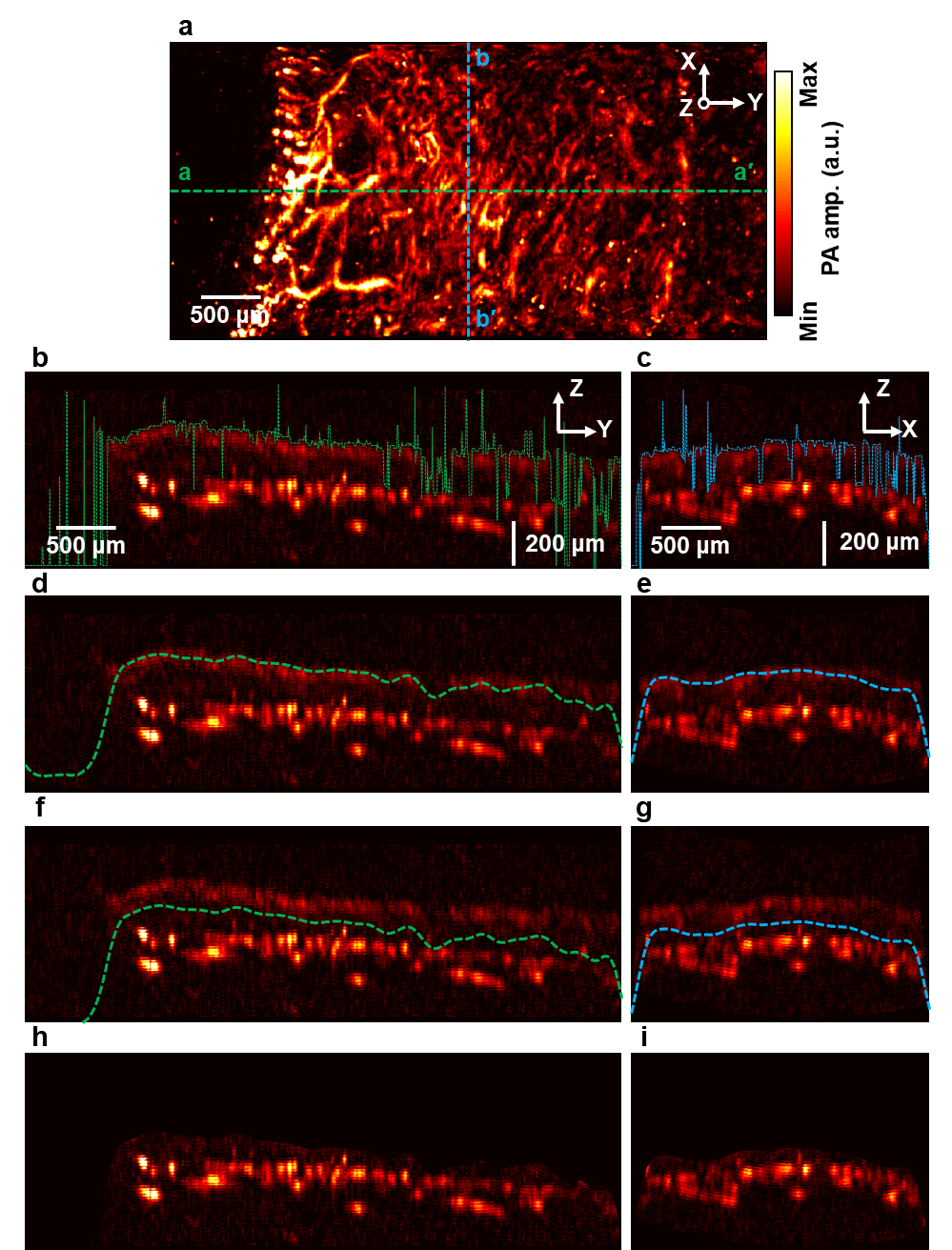


**Supplementary Fig. S6** Skin removal process. **a** PA MAP image of microstructures in a human cuticle *in vivo*. **b**, **c** Rough skin profiles and cross-sectional PA images of the planes indicated by the **b** green and **c** blue dashed lines (across a-a′ and b-b′, respectively) marked in **a**. **d**, **e** Smoothed skin profiles and equivalent PA images in **b** and **c**. **f**, **g** Dividing lines between skin signals and blood vessels signals, and equivalent PA images in **b** and **c**. **h**, **i** Skin-removed PA images. The upper signals of the dividing lines in **f** and **g** were all removed.


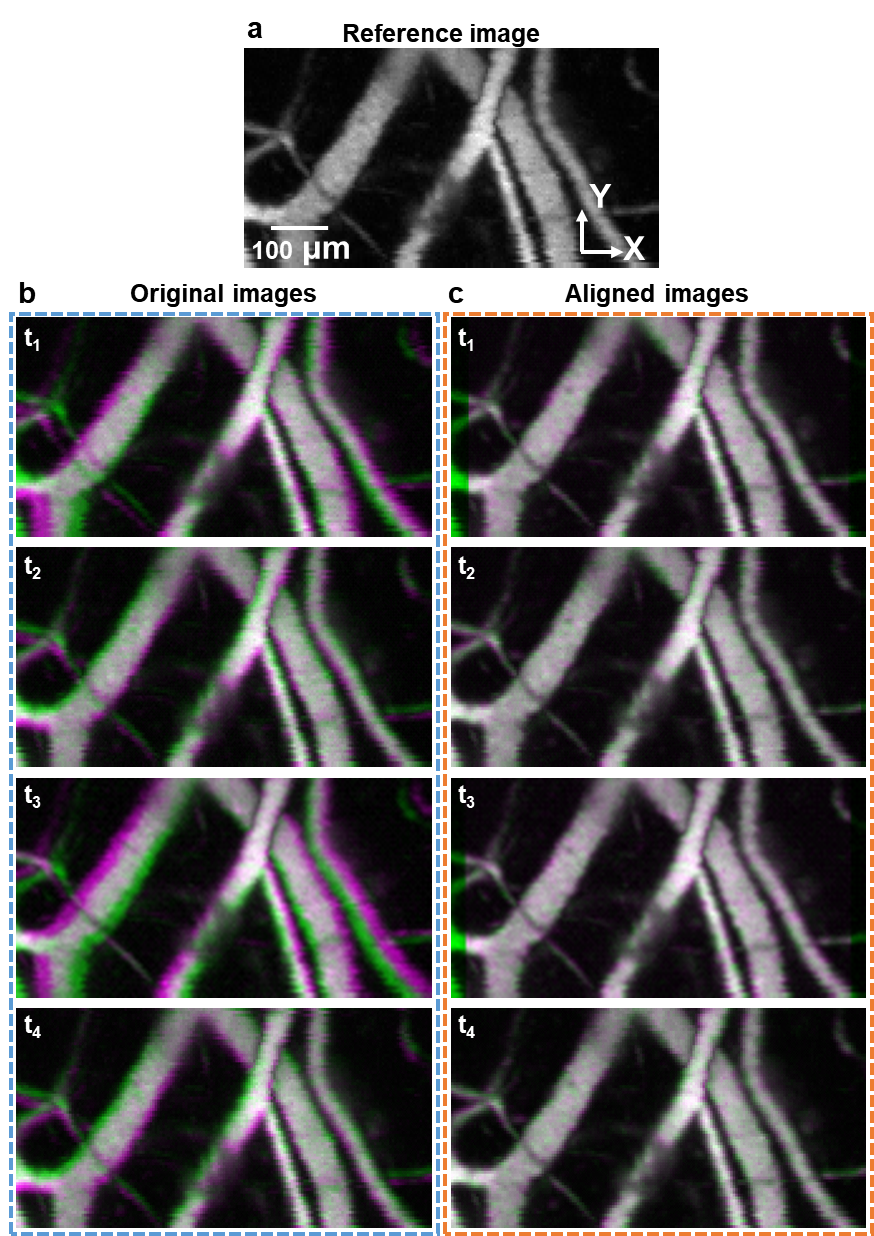


**Supplementary Fig. S7** Image registration. **a** Reference image. **b** Overlaid images of the reference image and unaligned images. **c** Overlaid image of the reference image and aligned images. In each overlaid image, the purple and green represent mismatched regions. Because the images taken at different times were not exactly aligned with each other, they were re-aligned to the reference image.


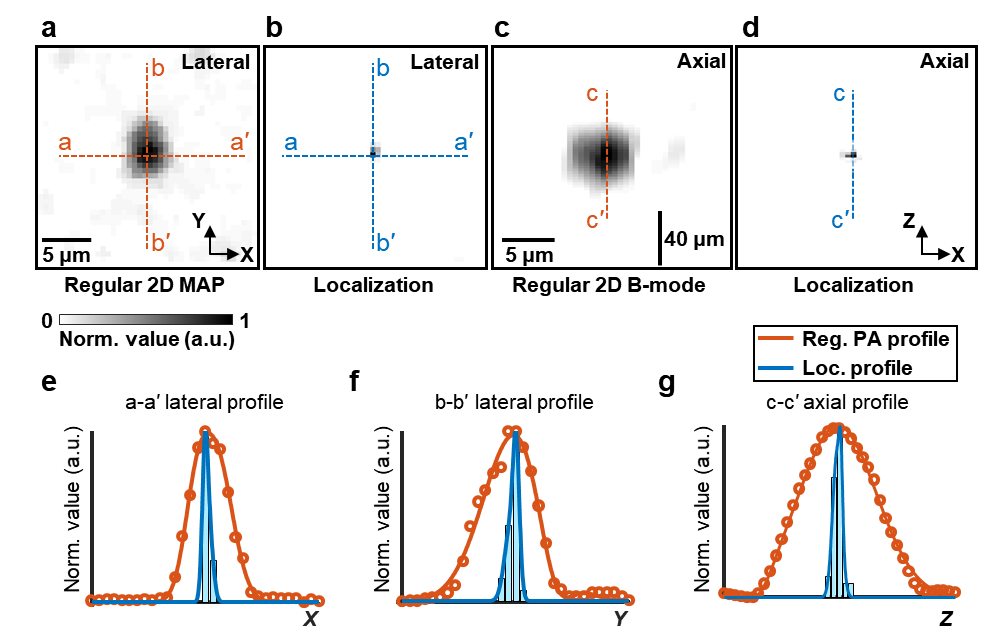
**Supplementary Fig. S8** Localization photoacoustic microscopy (PAM) of polystyrene particles *in vitro*. **a**, **c** Regular PA **a** MAP and **c** B-mode images of single polystyrene particle. **b**, **d** Localization PAM histogram images reconstructed by 3D histogram of the localized positions in a sequence of 100 image frames. The histogram images correspond to the **b** MAP image in **a** and the **d** B-mode image in **c**, respectively. **e**-**g** Profiles of PA signals and normalized histograms across **e** a-a′, **f** b-b′ and **g** c-c′ lines marked in **a**-**d**. The clear orange circles are for regular PA signal amplitudes. The orange curves are the fitted Gaussian curves of the normalized PA signals. The blue curves are the fitted Gaussian curves of the normalized histograms. MAP, maximum amplitude projection; PA, photoacoustic.


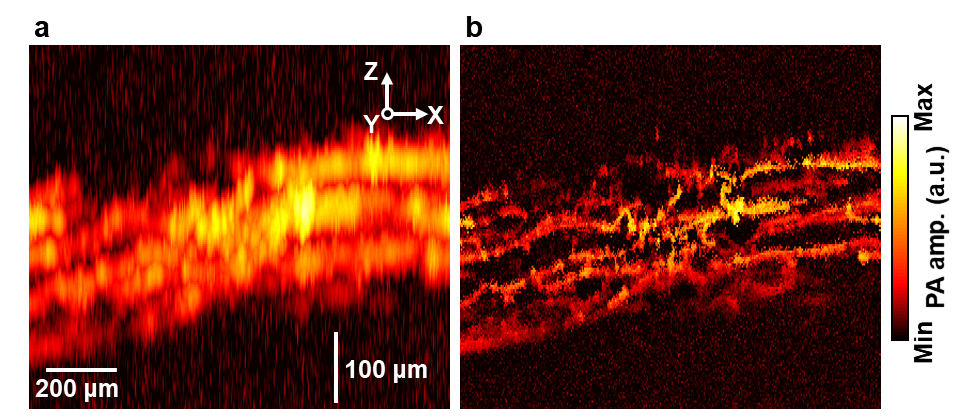


**Supplementary Fig. S9** *In vivo* **a** conventional and **b** localization PA MAP images of a mouse ear along the y-axis. The color bar is in a log scale. MAP, maximum amplitude projection; PAM; photoacoustic microscopy; PA, photoacoustic.

**
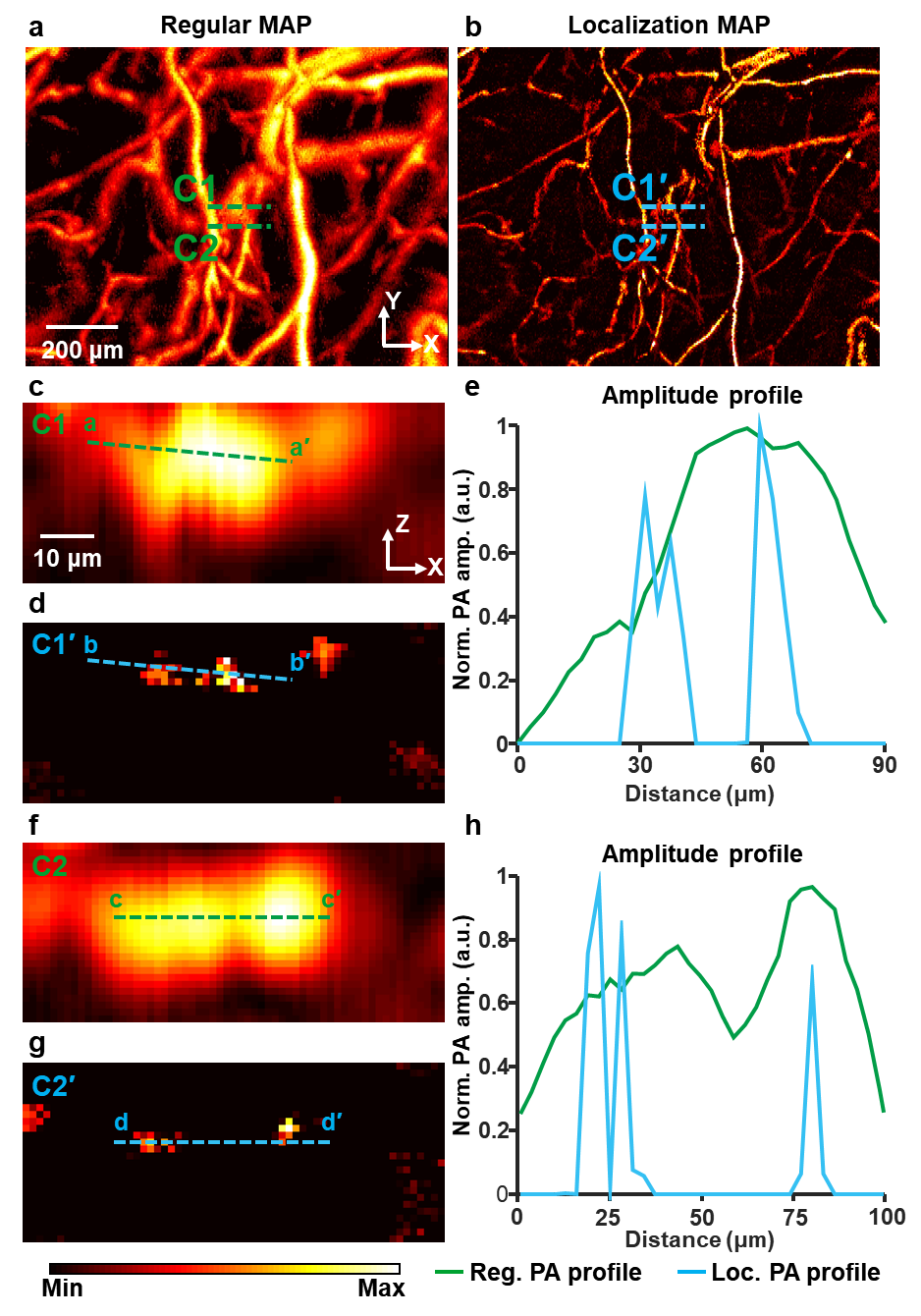
**

**Supplementary Fig. S10.** Quantification of the spatial resolution improvement with agent-free localization imaging *in vivo*. **a** Representative PA MAP image of a mouse ear. **b** Equivalent localization image. **c**, **d, f, g** Cross-sectional B-mode images of the region highlighted by the **c** C1, **d** C1′, **f** C2, **g** C2′ dashed line in **a** and **b**, respectively. **e** Profiles of the PA amplitude marked by the green dashed line a-a′ in **c** and the localization amplitude marked by the blue dashed line b-b′ in **d**. **h** Profiles of the PA amplitude marked by the green dashed line c-c′ in **f** and the localization amplitude marked by the blue dashed line d-d′ in **h**.

**Supplementary Reference**

1 Huang, B., Bates, M. & Zhuang, X. Super-resolution fluorescence microscopy. *Annual review of biochemistry* **78**, 993-1016 (2009).

2 Dean-Ben, X. L. & Razansky, D. Localization optoacoustic tomography. *Light: Science & Applications* **7**, 18004 (2018).

3 Hu, S., Rao, B., Maslov, K. & Wang, L. V. Label-free photoacoustic ophthalmic angiography. *Optics letters* **35**, 1-3 (2010).
